# Catalytic Conversion of Cellulose to Levulinic Acid by Metal Chlorides

**DOI:** 10.3390/molecules15085258

**Published:** 2010-08-02

**Authors:** Lincai Peng, Lu Lin, Junhua Zhang, Junping Zhuang, Beixiao Zhang, Yan Gong

**Affiliations:** State Key Laboratory of Pulp and Paper Engineering, South China University of Technology, Guangzhou 510640, Guangdong, China

**Keywords:** metal chlorides, cellulose, catalytic conversion, levulinic acid

## Abstract

The catalytic performance of various metal chlorides in the conversion of cellulose to levulinic acid in liquid water at high temperatures was investigated. The effects of reaction parameters on the yield of levulinic acid were also explored. The results showed that alkali and alkaline earth metal chlorides were not effective in conversion of cellulose, while transition metal chlorides, especially CrCl_3_, FeCl_3_ and CuCl_2_ and a group IIIA metal chloride (AlCl_3_), exhibited high catalytic activity. The catalytic performance was correlated with the acidity of the reaction system due to the addition of the metal chlorides, but more dependent on the type of metal chloride. Among those metal chlorides, chromium chloride was found to be exceptionally effective for the conversion of cellulose to levulinic acid, affording an optimum yield of 67 mol % after a reaction time of 180 min, at 200 °C, with a catalyst dosage of 0.02 M and substrate concentration of 50 wt %. Chromium metal, most of which was present in its oxide form in the solid sample and only a small part in solution as Cr^3+^ ion, can be easily separated from the resulting product mixture and recycled. Finally, a plausible reaction scheme for the chromium chloride catalyzed conversion of cellulose in water was proposed.

## 1. Introduction

**Figure 1:**
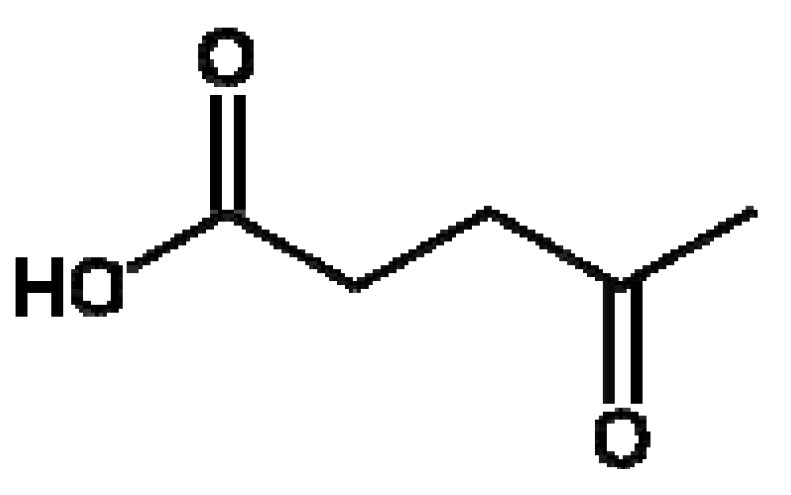
Structure of levulinic acid (LA).

Cellulose is abundantly available, and is considered as a promising alternative to non-renewable natural resources for the sustainable supply of fuel and chemicals in the future. Currently, extensive research is being carried out worldwide to identify and study chemical or biological transformation pathways to convert cellulose into biofuels and feedstock chemicals [1]. Among these, one attractive approach is the single-step conversion of cellulose to levulinic acid (LA, Figure 1) by acid-catalyzed hydrolysis. LA is a versatile building block containing a ketone carbonyl group and an acidic carboxyl group, which can be used for preparation of various high-value organic chemicals, polymers, resin, flavor substances, and fuel additives with numerous potential industrial applications [2,3].

To date, researchers have reported the conversion of biomass into LA using a dilute mineral acid such as HCl and H_2_SO_4_ as catalyst [4,5,6,7]. Although these hydrolysis reactions were effective, the use of the mineral acid causes serious pollution and promotes equipment corrosion and being difficult to recover from the reaction products for recycling [8]. Recently, solid acid catalysts have attracted considerable interest as heterogeneous catalysts which can overcome the above mentioned disadvantages of mineral acids in acid catalysis [9], but solid catalysts were found to display low catalytic activity in the conversion of indissoluble cellulose to LA in water due to the poor accessibility of the substrate. For instance, Wang *et al*. [10] used sulfated TiO_2_ as a solid acid catalyst to hydrolyze cellulose into LA, however, the highest yield achieved under their optimal experimental conditions was only 27.2 mol %. 

Metal salts are expected to show higher catalytic activity than other types of acid catalysts, with the additional possibility of being easy to be separate from the reaction products by supporting them on a carrier [11]. Several studies have reported that some metal salts can catalytically hydrolyze carbohydrates effectively into useful feedstock chemicals. Zhao *et al*. [12] studied the effect of a large number of metal chlorides on the conversion of sugars into 5-hydroxymethylfurfural (HMF) in ionic liquid solvents. Chromium (II) chloride was found to be uniquely effective, leading to the conversion of glucose to HMF in nearly 70% yield. Li *et al*. [13] presented an efficient strategy for CrCl_3_-mediated direct conversion of cellulose and glucose to HMF in ionic liquids under microwave irradiation. Besides the above reactions in ionic liquids that mainly yield HMF, reactions of carbohydrates with metal salts in aqueous solution and sub-critical water that chiefly produce LA and/or lactic acid have also been explored. For example, Efremov *et al*. studied the conversion of cellulose into LA in water using CoSO_4_, Fe_2_(SO_4_)_3_ and Al_2_(SO_4_)_3_ as catalysts [4]. The most active among the studied catalysts was Al_2_(SO_4_)_3_, which produced a LA yield of about 18 wt % at 250 °C. Rasrendra *et al*. reported the catalytic effect of a wide range of chloride and sulfate salts that were shown to affect the chemo-selectivity considerably in the conversion of D-glucose in aqueous solutions at 140 °C [14]. The main water-soluble product was lactic acid for Al (III), while HMF was formed in the highest yields by Zn (II). Lu *et al*. [15] determined glucose and HMF decomposition kinetics catalyzed with some metal chlorides in liquid water at high temperatures by using glucose as representative model for cellulose. The dominating product in the conversion of carbohydrates catalyzed by metal salts in sub-critical water (*T* = 200–360 °C) appears to be lactic acid, according to Bicker *et al*. [16] and Kong *et al*. [17]. However, there are only a few reports on the effects of some types of metal chlorides catalyzing conversion of cellulose to LA in terms of the composition and yield of products. Seri *et al*. [11] used lanthanum (III) chloride as catalyst during the conversion of cellulose in water at 250 °C and identified the products, but the yield of LA was reported to be low. Therefore, further studies are still necessary to understand the catalytic mechanisms of metal chlorides and find more reactive catalysts for the conversion of cellulose to LA.

The objective of this study was to examine the catalytic performances of twelve common metal chlorides, including alkali metals (Li, Na and K), alkaline earth metals (Mg and Ca), transition metals (Cr, Mn, Fe, Co, Cu and Zn) and a group IIIA metal (Al), on the conversion of cellulose to LA in hot-compressed water. Among those metal chlorides, CrCl_3_ was found to be uniquely effective. Based on its use, we systematically sought to optimize the LA yield by altering the process conditions (agitation speed, reaction time, reaction temperature, catalyst dosage and substrate concentration) and propose the possible catalytic scheme of the metal chlorides such as CrCl_3_ for the conversion of cellulose to LA.

## 2. Results and Discussion

### 2.1. Catalytic Effects of Metal Chlorides on the Conversion of Cellulose

**Figure 2:**
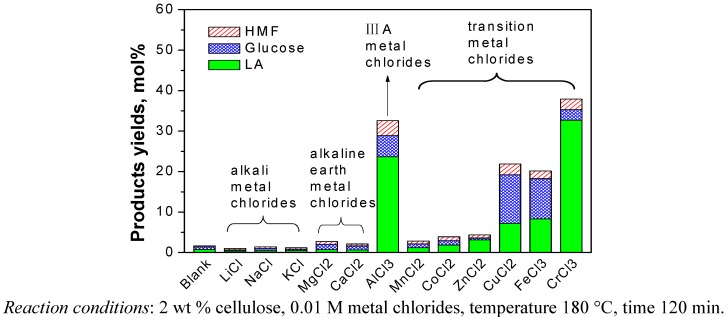
Catalytic effects of metal chlorides on the conversion of cellulose.

**Figure 3:**
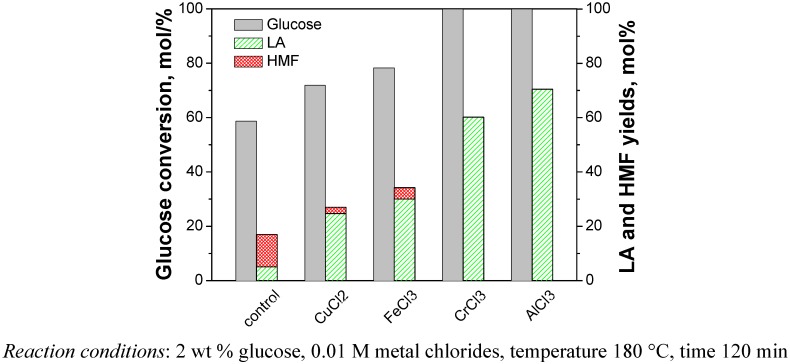
Catalytic effects of metal chlorides on the conversion of glucose.

The cellulose was reacted at 180 °C for 120 min with 0.01 M of metal chlorides in water. After the reaction, the target products (*i.e.*, LA and formic acid) as well as the main intermediates (*i.e.*, glucose, fructose and HMF) in the liquid samples were analyzed. The glucose, HMF, LA and FA were detected for all samples using various types of metal chlorides as catalyst. Fructose was in trace amounts or could not be detected. This is due to the faster dehydration of fructose to HMF than that of glucose, which already been shown in previous studies [18,19]. Other than the products detected, there were probably several other compounds in the liquid samples. For instance, Rasrendra *et al*. showed that glycolic acid, acetic acid and lactic acid were also observed for the conversion of glucose in water at 140 °C using transition metal and aluminum salts [14]. These substances were not checked here, because the objective of this paper was to produce LA. The yields of main products including glucose, HMF and LA with and without the addition of metal chlorides as catalyst during the conversion of cellulose are compared in Figure 2. The results showed that alkali and alkaline earth metal chlorides did not lead effectively to the conversion of cellulose, and there was no significant difference with the blank (no catalyst). By contrast, several transition metal chlorides (CrCl_3_, FeCl_3_ and CuCl_2_) and the group IIIA metal chloride (AlCl_3_) showed significantly higher activity than the blank reaction and the yields of LA for these metal chlorides decreased in the order CrCl_3 _> AlCl_3 _> FeCl_3 _> CuCl_2_ under identical operating conditions. However, the experiment with FeCl_3_ and CuCl_2_ as catalyst had high the residual amounts of glucose in the products, which indicated that the FeCl_3_ and CuCl_2_ are unfavorable for further converting glucose into LA. In order to further prove the catalytic effect of these metal chlorides including CrCl_3_, AlCl_3_, FeCl_3_ and CuCl_2_, we also conducted experiments with glucose instead of cellulose under the same reaction conditions (Figure 3). 

It showed that these metal chlorides have similar activity for the conversion of glucose compared with that of cellulose. FeCl_3_ and CuCl_2 _as catalyst during the conversion of glucose still left some amount of residual glucose in the resulting products. On the other hand, glucose was totally consumed and the yields of LA were very high when CrCl_3_ and AlCl_3_ were used as catalyst. A similar effect of the type of salts on the glucose conversion was also shown in Rasrendra’s work [14], but the glucose conversion there was somewhat low. Furthermore, either lactic acid or HMF was the major product, and the amount of LA was relatively low. This is because the reaction temperature used in their research was low at 140 °C. Lower temperature is disadvantageous to product LA [7], which was also confirmed in the following experiments. However, different from the case of cellulose, AlCl_3_ showed higher yield of LA and better selectivity for the conversion of glucose than CrCl_3_. Based on these results it was concluded that AlCl_3_is more favorable for promoting the isomerization of glucose to fructose, whereas CrCl_3 _had higher activity than AlCl_3 _for the hydrolysis of cellulose. Cellulose was rapidly depolymerized in the presence of CrCl_3_ so that it was more easily converted to glucose, and then to LA.

### 2.2. Relationship between Reactivity and Acidity of Reaction System

**Figure 4:**
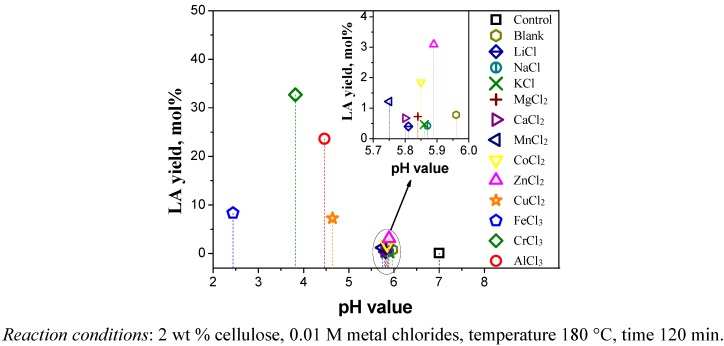
Relationship between LA yield and initial pH value of reaction system with 0.01 M metal chlorides measured at room temperature.

**Figure 5:**
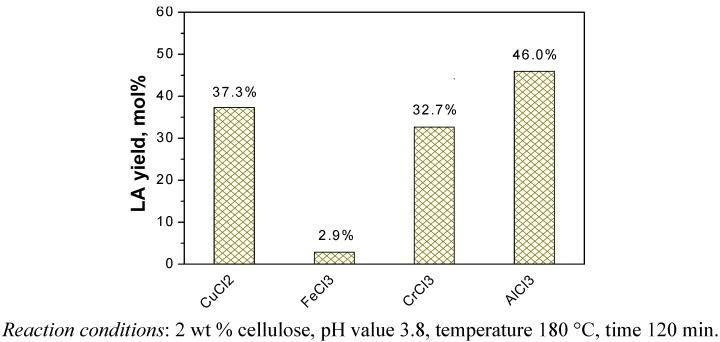
Effect of various types of metal chlorides on the hydrolysis of cellulose at the same initial pH value.

Many metal chlorides are strong Lewis acids, and in water they may undergo hydrolysis to form basic salts, resulting in the pH value of the solution becoming quite acidic. Figure 4 shows the relationship between LA yield and initial pH value of the reaction system measured at room temperature with 0.01 M metal chlorides. It is particularly interesting to note that the solutions of metal chlorides including CrCl_3_, AlCl_3_, FeCl_3_ and CuCl_2_ with high activity in the conversion of cellulose to LA were quite acidic, showing lower initial pH values in the reaction systems. The other added metal chlorides showed very slight variation in the initial pH value of reaction system in comparison with the blank (without the addition of metal chlorides), and did not work for the conversion of cellulose. The water solution for a blank experiment exhibited weak acidity, which is due to dissolved carbon dioxide. For this reason, a control experiment was also carried out at an intermediate pH value adjusted by sodium hydroxide. It can be seen from Figure 4, the yield of LA for the control reaction was almost the same as that of the blank. These finding suggested that a certain amount of acidity in the initial reaction system was essential for the production of LA. However, this did not mean that the lower initial pH value was closely related to the higher the yield of LA. For example, the initial pH value of reaction system with FeCl_3_ was lower than that with CrCl_3_ and AlCl_3_, but the yield of LA was lower. Accordingly, we believed catalytic performance depends mainly on the type of metal chloride used, rather than the acidity of the solution. To verify the conclusions, the same initial pH value of the reaction system with various metal chlorides was achieved by changing the amount of metal chlorides. As shown in Figure 5, it can be seen that different types of metal chlorides as catalyst produced significantly different yields of LA at the same initial pH value. The results further demonstrated the types of metal chlorides play a major role in the cellulose hydrolysis and glucose dehydration.

### 2.3. Effects of Reaction Conditions on Yields of Products

Among the metal chlorides tested as catalyst, CrCl_3_ was found to be uniquely effective in the conversion of cellulose to LA. Hence, it was chosen as the most suitable catalyst for further exploration. To obtain the highest possible LA yield, it is necessary to optimize the reaction conditions by varying the speed of agitation, reaction time, reaction temperature, catalyst dosage and substrate concentration, and the results are discussed in the following sections.

#### 2.3.1. Agitation Speed

**Figure 6:**
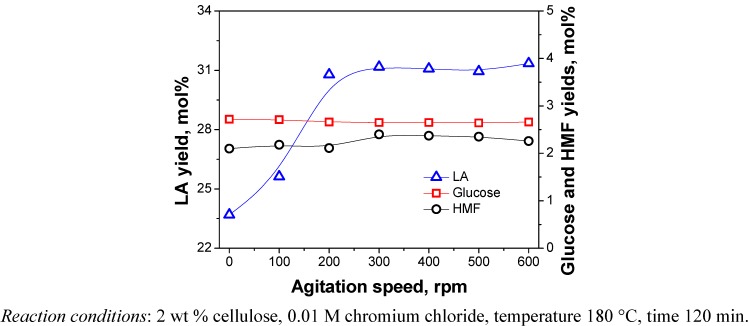
Effect of agitation speed on yields of products.

The solid-liquid phase catalytic reaction system between the cellulose and the water solution of metal chlorides may suffer from severe mass transfer limitations that affect the apparent reaction rate. Increasing the agitation speed might increase the contact area of the two phases, and thus remove the interfacial mass transfer resistance [20]. The reaction was conducted by changing the agitation speed from 0–600 rpm and the results are given in Figure 6. 

As it can be observed from the figure, the yield of LA increased rapidly as agitation speed increased from 0 rpm to 200 rpm, and then remained almost constant. However, there was no clear influence of the agitation speed on the yields of glucose and HMF. These results indicated that the interfacial mass transfer resistance between the cellulose surface and liquid phase was negligible when the agitation speed was above 200 rpm. The conclusion also proved that the frontal experimental results with agitation speed of 300 rpm were an intrinsic kinetic feature for the good conversion of cellulose to LA.

#### 2.3.2. Reaction Time

**Figure 7:**
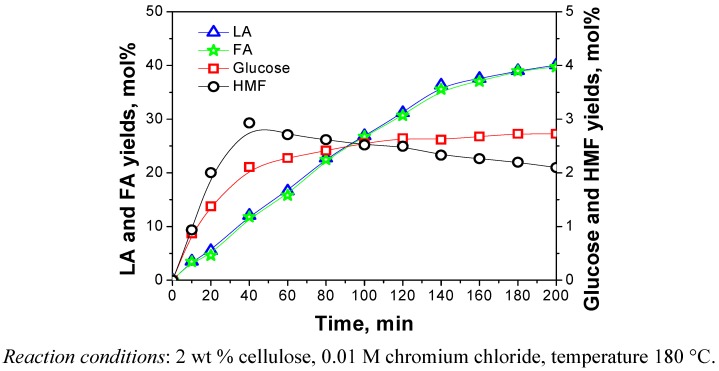
Time courses of the products during CrCl_3_-catalyzed hydrolysis of cellulose.

**Scheme 1:**
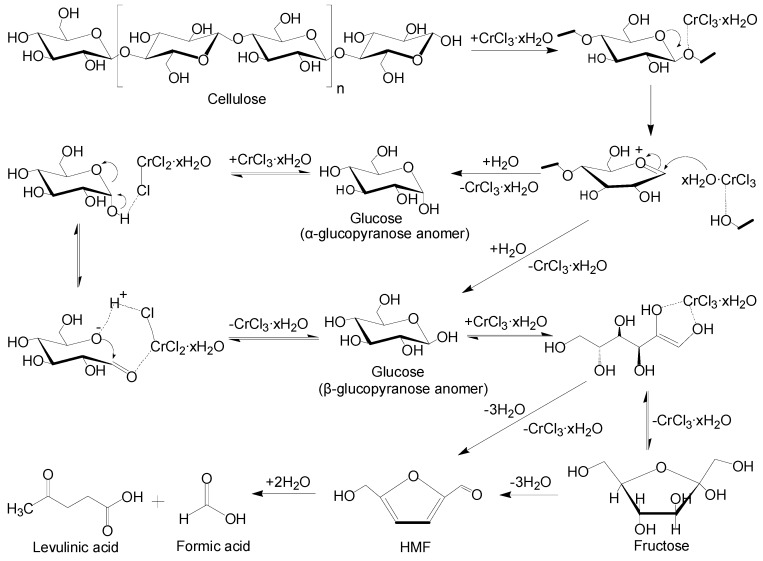
Proposed mechanism for the CrCl_3_ catalyzed conversion of cellulose to LA in water.

Time courses of the main water-soluble products during CrCl_3_-catalyzed hydrolysis of cellulose were as shown in Figure 7. It seems that with the prolonging of reaction time, the amount of glucose reached a low constant level within a very short period of time. The yield of HMF, being slightly higher during the initial stage of the reaction and then decreased, was kept very low throughout the reaction. The amount of LA grew very fast for the first 140 min. Then it was observed slightly to rise after 140 min. It was found that 180 min was sufficient for completion of the hydrolysis reaction. The time course of the amount of formic acid (FA) was similar to that of LA. In addition, LA and FA were always formed in a 1:1 molar ratio in line with the reaction stoichiometry given in Scheme 1. The result was also proved in previous research [6]. This finding indicates that LA and FA are stable under the reaction conditions employed and do not decompose to other products.

#### 2.3.3. Reaction Temperature

**Figure 8:**
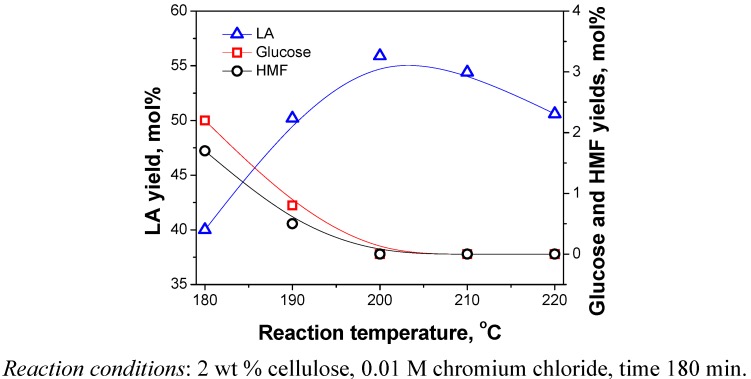
Effect of reaction temperature on yields of products.

The effect of reaction temperature on the cellulose conversion into LA was investigated, and the experiments were conducted at 180, 190, 200, 210 and 220 °C, respectively. Figure 8 shows the effect of the temperature on the yields of products. It can be observed that temperature has a significant effect on LA yield. When the reaction temperature was increased from 180 to 200 °C, there was a significant increase in the yield of LA and the amount of glucose and HMF were gradually decreased. When the reaction temperature rose above 200 °C, the yield of LA began to fall, and intermediates such as glucose and HMF weren’t detected. Higher temperatures thus could accelerate the rate of chemical reactions, but unwanted side reactions also appeared at the same time. For example, LA is unstable above 230 °C, which would be dehydrated to unsaturated lactones [7]. Additionally, more humins may occur during the reaction. Therefore, elevation of temperature is unfavorable for the extent of reaction, thus the reaction temperature was set to 200 °C in this experiment.

#### 2.3.4. Catalyst Dosage

**Figure 9:**
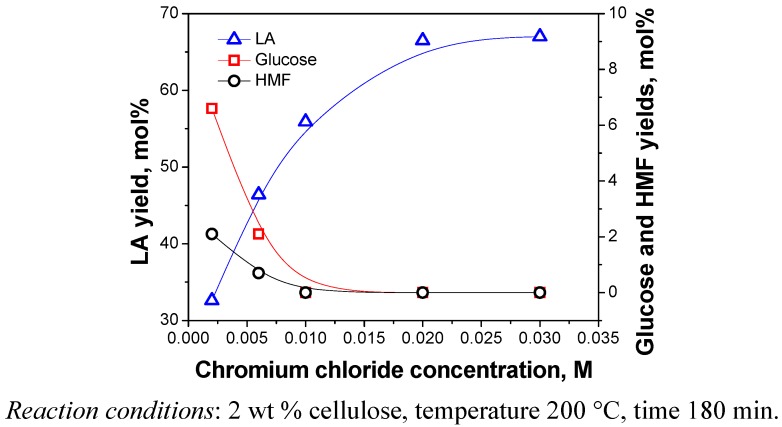
Effect of catalyst dosage on yields of products.

The effect of catalyst dosage on the yields of products is shown in Figure 9. The concentration of CrCl_3_ into the reaction mixture was varied from 0.002 M to 0.03 M. As can be seen, catalyst dosage significantly affected the cellulose conversion. The effects on glucose and HMF were similar to those in Figure 8. The yield of LA increased rapidly with increasing catalyst dosage to an optimum condition, and then did not produce a corresponding increase with a further increase Addition of excess catalyst has little effect. Similar results have also been observed for LA production catalyzed by other catalysts such H_2_SO_4_ and HCl [2,5]. Accordingly, when catalyst dosage meets the requirements for the reaction, no more would be needed. Taking the cost and the efficiency into considerations, the most beneficial concentration of CrCl_3_ was chosen to be 0.02 M.

#### 2.3.5. Substrate Concentration

**Figure 10:**
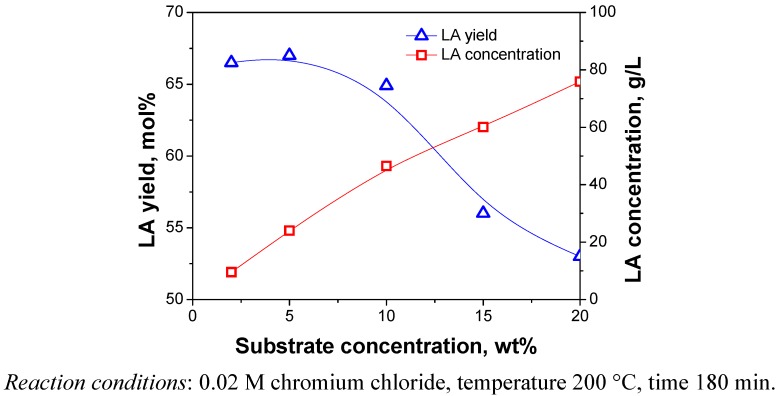
Effect of substrate concentration on concentration and yield of LA.

The optimal concentration of substrate is very important for the efficient use of cellulose and the final LA concentration. Figure 10 shows the influence of the substrate concentration with respect to the LA concentration and LA yield. Higher LA yield was achieved at lower substrate concentration, but the LA concentration was low, as shown in Figure 10. A higher LA concentration is extremely favorable that can not only cut down energy consumption of the purification of LA, but also reduce the amount of wastewater produced. When catalyst dosage was fixed, increasing substrate concentration resulted in more cellulose available for conversion, which means higher LA concentration in the reaction samples, but the yield of LA was found to decrease markedly from 10 wt % to 15 wt %. The reason is probably due to product feedback inhibition, reactivity diminution or insufficient catalyst dosage for the additional cellulose. Therefore, compromises have to be made between concentration and yield of LA for economic reasons.

### 2.4. Metal Tracing and Recycling

**Figure 11:**
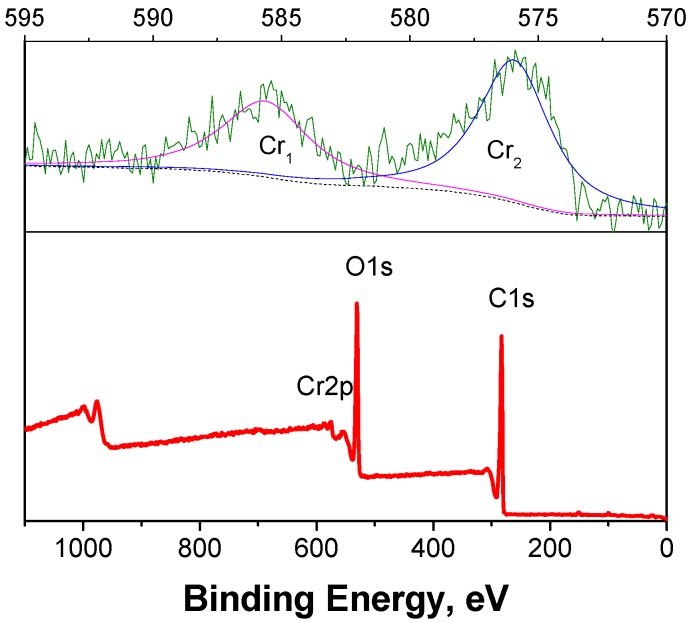
XPS survey spectrum of solid product and high-resolution spectrum of Cr 2p.

The dosage of CrCl_3_(0.02 M) during the production of LA in this paper was less compared with that of H_2_SO_4_ (0.1–1 M) [2,5,6] and HCl (~1.2 M) [7] in the previous literature, but the disposal of the catalysts will not only waste resources, but also cause environmental pollution, so it is extremely important that the chromium metal be recycled. Here, the existing forms and the separation of chromium metal from the reaction product were investigated. The amount of chromium ion in the liquid product was determined using atomic absorption spectrometry. The results show that it was significantly lower, with only 30% of that measured before the reaction. It is easy to separate by supporting it on a carrier. This finding also indicates that the existing form of most chromium has changed from the Cr^3+^ ion found in solution. During the reaction, some dark-brown insoluble substances were observed. Previous studies have already shown that the substances known as humins are formed from the side-reactions of the acid-catalyzed decompositions of cellulose, glucose and HMF [6,21]. The presence of the solid products after the reaction was confirmed by XPS analysis, as shown in Figure 11. Inspection of the XPS survey spectrum revealed that C and O are the predominant species, occuring at 283.19 eV and 531.02 eV, respectively, and they are mainly derived from humins. In addition, chromium was also detected, but chlorine did not appear. This suggests that most of the chromium metal may be adsorbed in the solid products and no longer existed as chloride. High-resolution scanning of the XPS spectrum of Cr 2p with its characteristic peaks at 585.62 eV of Cr_1_ and 575.93 eV of Cr_2_, respectively, are also presented in Figure 11. 

**Figure 12:**
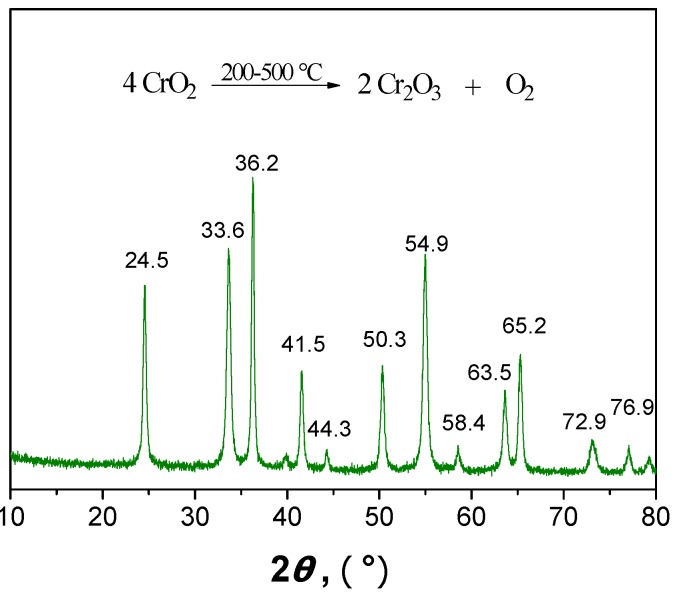
XRD pattern of solid product calcined at 400 °C for 8 h.

The chromium peaks might be caused by some its oxides, Cr_1_ of Cr_2_O_3_ and Cr_2_ of CrO_2_, which are known to be existing forms of chromium in the solid product. The Cr_1_ to Cr_2_ peak intensity ratio was found to be of 0.65. They can be formed during the reaction process because of high temperature and pressure. For separating the chromium oxides from the humins, the solid product was calcined at 400 °C for 8 h in static air. It was observed that the color of solid product changed from dark-brown to green. The humins were thus removed from the solid product. The XRD pattern of the resulting product was shown in Figure 12. 

The resulting product was found to display some well distinguished peaks in the 2*θ* range from 10° to 80°. These peaks were all assigned to Cr_2_O_3_. This is because chromium dioxide also breaks down into chromium trioxide and oxygen under high temperature condition. Cr_2_O_3_ is not only a raw material for Cr production but also of possible use for the preparation of refractory ceramics and green pigments.

### 2.5. Mechanism of Catalytic Degradation

The conversion of cellulose to LA is a complex multistep process. It is generally believed that the polymer chains of cellulose are broken down into low molecular weight fragments and finally to glucose. Then, the glucose is decomposed to HMF, which is further converted into LA and FA [6]. All above-mentioned products were detected in this study. Some metal chlorides have shown obvious catalytic effects for the conversion of cellulose and glucose in many studies, but it is still unclear how metal chlorides affect the reaction process. In particular, the exceptional effectiveness of CrCl_3_ in the conversion of cellulose not only reflected in our study for the LA production in water, but also in Li’s work for the HMF production in ionic liquid [13]. It is likely that CrCl_3_ system have very strong coordinating abilities with certain kinds of groups in the reactants. Li believed that CrCl_3_ in [C_4_mim]Cl form [C_4_mim]_n_[CrCl_3+n_] complexes and coordination chemistry involving CrCl_3_ played a predominant role for cellulose hydrolysis and glucose dehydration. Therefore, according to these findings and results from our experiment, it could be induced that the catalytic reaction mechanism of CrCl_3_ in water could be similar to that in ionic liquids. A plausible reaction mechanism for the CrCl_3_ catalyzed conversion of cellulose to LA in water is thus proposed, as shown in Scheme 1. 

The CrCl_3_ could form hydrated complexes in aqueous solution. In this scheme, the cellulose hydrolysis step and the coordination of the glycosidic oxygen of cellulose with chromium, which is acting as the Lewis acid, would help the breakdown of the glycosidic linkages, with the coordinated water molecules from the hydrated CrCl_3_ participating as a nucleophile to form glucose, similar to what was proposed for the ZnCl_2_-mediated degradation of cellulose to glucose reported by Amarasekara *et al*. [22]. In the glucose degradation process, the hydrated complex of CrCl_3_ facilitated mutarotation of the α-anomer of glucose to the β-one through hydrogen bonds of chloride anions with the hydroxyl groups. Then, the hemiacetal portion of β-glucopyranose forms a Cr (III) enolate anion complex that leads to isomerization of glucose to fructose, which would be simultaneously dehydrated to HMF. HMF is unstable in water and could be further converted into LA and FA under the reaction condition.

## 3. Experimental

### 3.1. Materials

The twelve kinds of metal chlorides used in this study were lithium chloride (LiCl), sodium chloride (NaCl), magnesium chloride (MgCl_2_), potassium chloride (KCl), calcium chloride (CaCl_2_), manganese chloride (MnCl_2_), cobalt chloride (CoCl_2_), zinc chloride (ZnCl_2_), ferric chloride (FeCl_3_), copper chloride (CuCl_2_), chromium chloride (CrCl_3_), and aluminium chloride (AlCl_3_) from Kermel (Tianjin, China). Microcrystalline cellulose (DP~200) with an average particle size of 100 μm was obtained from Sinopharm Chemical Reagent (Shanghai, China). The above reagents and chemicals were all of analytical grade and used without further purification or treatment. Glucose, fructose, 5-hydroxymethylfurfural, levulinic acid, and formic acid used for calibration, with purity of over 99%, were purchased from Aladdin Reagent (Shanghai, China). Deionized water was used for all reactions.

### 3.2. Equipment and Procedure

The hydrolysis experiments were carried out in a cylindrical stainless steel pressurized reactor with inner diameter 52 mm, depth 118 mm and 250 mL total volume. The reactor was heated in an adjustable electric stove. The temperature of the reactor contents was monitored by a thermocouple connected to the reactor. For each experiment, a weighed amount of microcrystalline cellulose or glucose, water and the desired metal chlorides were mixed to form a suspension and put into the reactor, which was then brought to the desired temperature by external heating. A typical reaction procedure was as follows: cellulose (2 g) and water (100 mL) were introduced into the reactor along with 0.001 mol chromium chloride. The reactor was heated at 180 °C for 120 min and shaken at 300 rpm. After the reaction, the reactor was taken from the stove and quenched in an ice cool water bath to terminate the reaction.

### 3.3. Sample Analysis

The samples taken from the reactor were filtered and washed with a portion of deionized water to recover the liquid and solid products. The amount of products in the liquid samples was quantitatively analyzed by ion chromatography (Dionex ICS-3000, USA) after appropriate dilution with pure water. A CarboPac PA1 (2 mm × 250 mm) analytical column with electrochemical detection was employed to detect the glucose and HMF. A solution of sodium hydroxide (100 mM) was used as the eluent with a volumetric flow rate of 0.325 mL/min at 30 °C. An IonPac AS11-HC (4 mm × 250 mm) analytical column with conductivity detection was employed to detect the LA and FA. A solution of sodium hydroxide (10 mM) was used as the eluent with a volumetric flow rate of 1.0 mL/min at 30 °C. The sample injection volume was 20 μL. The amount of each compound in the liquid products was determined using calibration curves obtained by analyzing standard solutions with known amounts. The yield of products on a molar base was calculated using the following equation [23]:

Product yield (mol %) = 100M/N

where N is total amount (mol) of glucose monomer in cellulose and M denoted the amount (mol) of products after reaction. The sample was further analyzed when CrCl_3_ was used as a catalyst. The amount of chromium ion in the liquid product was determined using atomic absorption spectrometry (Hitachi Z-2000, Japan). The elemental composition of the solid product was analyzed with X-ray photoelectron spectroscopy (XPS). XPS measurement was performed on a Kratos Ultra system with 1 eV per step for survey spectrum over a binding energy range of 0–1,100 eV. High-resolution spectrum of Cr 2p was an average of five scans acquired at pass energy of 20 eV and resolution of 0.1 eV per step. X-ray diffraction (XRD) analysis of the solid product calcined at 400 °C for 8 h was carried out using Bruker D8 X-ray diffractometer with Cu Ka radiation operated at 40 kV and 40 mA. Data was collected from 2*θ* between 10° and 80° in a step of 0.02°/s.

## 4. Conclusions

In this study it was shown that different types of metal chlorides have markedly different catalyst effects on the conversion of cellulose. Some transition metal chlorides (such as CrCl_3_, FeCl_3_ and CuCl_2_) and a group IIIA metal chloride (AlCl_3_) showed higher catalytic activity for the reaction. The catalytic performance was correlated with the acidity of the reaction system due to addition of the metal chlorides, but more dependent on the types of metal chlorides. The reaction time, reaction temperature, catalyst dosage and substrate concentration during the degradation of cellulose play significant roles on the production of LA. The highest yield of LA was found to be 67 mol % when CrCl_3_ was used as catalyst. An advantage of using CrCl_3_ as catalyst is that less is used. Additionally, chromium metal, most of which was present in its oxide form in the solid products and only a small part in solution as Cr^3+^ ion, can be easily separated from the reaction products and recycled.
